# A new marker for predicting sentinel lymph node metastasis in early (cT1-2N0) breast cancer: Tumor-infiltrating lymphocytes (TILs)

**DOI:** 10.1371/journal.pone.0320487

**Published:** 2025-03-19

**Authors:** Xihao Ni, Weitao Wang, Huimin Sun, Ran An, Ying Lei, Chang-liang Wang

**Affiliations:** 1 School of Clinical Medicine, Shandong Second Medical University, Weifang, Shandong Province, People’s Republic of China,; 2 Department of Pathology, Weifang People’s Hospital, Weifang, Shandong Province, People’s Republic of China,; 3 Department of Breast Surgery, Weifang People’s Hospital, Weifang, Shandong Province, People’s Republic of China; Sun Yat-Sen University, CHINA

## Abstract

**Background:**

Tumor-infiltrating lymphocytes (TILs) are associated with lymph node metastasis and prognosis in breast cancer. Therefore, we explored the value of TILs in predicting sentinel lymph node metastasis (SLNM) in patients with early-stage (cT1-2N0) breast cancer and provided a new method for preoperative assessment of SLNM status.

**Methods:**

This study included 337 patients with early-stage breast cancer who underwent surgery at our hospital from January 2022 to December 2023. The expression of estrogen receptor (ER), progesterone receptor (PR), human epidermal growth factor receptor 2 (HER2), and Ki-67 in the patients was assessed using immunohistochemistry (IHC). TILs in the core needle biopsy samples were evaluated histopathologically, and patients were divided into high and low TILs groups based on the density of TILs. Statistical analysis was conducted, and a predictive model was established.

**Results:**

The study found that patients with high TILs had a significantly lower rate of SLNM compared to those with low TILs (P < 0.001). The cT stage and the level of TILs were identified as independent predictive factors for SLNM. The ROC curve analysis indicated that the density of TILs has good predictive efficacy for SLNM. Based on the results of the multivariate regression analysis, a nomogram predictive model for SLNM was constructed.

**Conclusions:**

Our study showed that the density of TILs and cT stage are independent predictive factors for SLNM in early-stage (cT1-2N0) breast cancer, and the predictive effect of TILs density on SLNM is significant in Luminal and triple-negative breast cancers.

## 1. Introduction

Breast cancer is one of the most common malignant tumors among women worldwide, and its incidence rate ranks first, with patient prognosis closely related to tumor staging [1]. Therefore, early diagnosis and treatment of breast cancer are crucial. In the era of precision medicine, the treatment of breast cancer is shifting towards a more precise, minimally invasive, and personalized approach. Sentinel lymph node biopsy (SLNB), as a method to predict the status of axillary lymph nodes, has been used in breast cancer since 1994 [2]. The traditional view is that patients with positive sentinel lymph nodes require Axillary Lymph Node Dissection (ALND), however, ALND may lead to lymphedema and functional impairment of the ipsilateral upper limb, which affects the quality of life of patients. In recent years, clinical trials such as Z0011, AMAROS, and the most recent SENOMAC have shown that breast cancer patients with micrometastasis or 1-2 positive SLNs in cT1-3cN0 can forego ALND [3–5]. In addition, some studies have suggested that sentinel lymph node biopsy itself may be an over-treatment for patients with clinically negative axillary lymph nodes [6]. A recent SOUND randomized clinical trial showed that breast cancer patients with negative axillary lymph node ultrasound findings can safely avoid any axillary surgery for cT1 [7]. Therefore, it is of great significance to find early indicators for predicting SLNM in patients with clinically negative axillary lymph nodes. Immune cells in the tumor microenvironment generally include T lymphocytes, B lymphocytes, natural killer cells, tumor-associated macrophages (TAMs), dendritic cells, and myeloid-derived suppressor cells, etc. [8,9]. TILs are a group of lymphocytes present within and in the stroma of the tumor nest, which can directly reflect the state of the tumor immune microenvironment and play an important role in the occurrence, progression, and control of tumors [10,11]. It is worth noting that there have been no reports on the relationship between SLNM and TILs in early-stage (cT1-2N0) breast cancer. The application of TILs in early breast cancer provides a new perspective for the formulation of individualized treatment strategies. By detecting the infiltration level of TILs, it is possible to more accurately predict the metastasis of sentinel lymph nodes, thereby enabling the formulation of more personalized treatment plans for patients, avoiding unnecessary ALND, and reducing complications for patients. Therefore, TILs play an important role in the prediction of sentinel lymph node metastasis in early breast cancer, with significant novelty and clinical significance. Through the detection and analysis of TILs, new evidence can be provided for the formulation of individualized treatment strategies, optimizing clinical decision-making, improving treatment outcomes and patient survival rates, while also reducing complications and medical costs. This study aims to explore the value of TILs in predicting SLNM in early-stage (cT1-2N0) breast cancer patients and to provide a new method for preoperative assessment of SLNM status.

## 2. Materials and methods

### 2.1. Patients

For research purposes, we began collecting patient information on May 1, 2024, and authors had access to information that could identify individual participants during or after data collection. We collected patients who were preoperatively diagnosed with early-stage (cT1-2N0) breast cancer and underwent surgery as first-line treatment at our hospital from January 2022 to December 2023. Inclusion criteria were: (1) female patients with invasive breast cancer confirmed by core needle biopsy pathology before surgery; (2) clinical staging of cT1-T2N0 for breast cancer patients; (3) no anti-cancer treatment of any form before surgery, including neoadjuvant chemotherapy, neoadjuvant endocrine therapy, neoadjuvant targeted therapy, and neoadjuvant radiotherapy; (4) complete clinical and pathological data; (5) the HE-stained sections from the core needle biopsy of the breast tumor were stored in the pathology department of our hospital and were available for TILs counting. Exclusion criteria were: (1) core needle biopsy of the breast tumor only showing in situ carcinoma components, with or without microinvasion; (2) stage IV metastatic breast cancer; (3) bilateral breast cancer; (4) confirmed to have other malignant tumors at the time of breast cancer diagnosis. Based on the inclusion and exclusion criteria, a total of 337 patients were ultimately included.

### 2.2. Basic data

**Fig 1:**
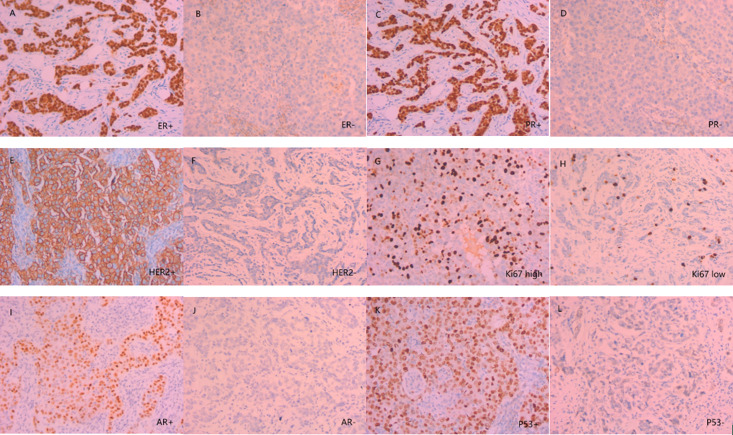
Immunohistochemical test images (×200). A: ER+; B:ER-; C: PR+; D:PR-; E: HER2+; F: HER2-; G: Ki67 high; H: Ki67 low; I: AR+; J: AR-; K: P53+; L: P53-.

The clinical data of the patients were collected using our hospital’s electronic medical record system, including age, menstrual status, tumor location, and cT stage, among others. IHC was used to detect the expression of ER, PR, HER2, and Ki67 in the biopsy tissue. According to the 2010 ASCO/CAP guidelines [12], staining of ER and PR at more than 1% was considered positive. HER2 expression was measured according to the 2013 ASCO/CAP guidelines [13], where HER2 positivity was defined as +++ on IHC or gene amplification on fluorescence in situ hybridization (FISH) for IHC ++. Based on previous studies, a Ki67 >  14% was considered a high proliferation index, and Ki67 ≤  14% was considered a low proliferation index [14,15] (Fig 1). All cases were classified into Luminal A (ER+, PR+ /-, HER2-, low Ki67), Luminal B (ER+, PR+/-, HER2+ or ER+, PR+/-, HER2-, high Ki67), HER2 enriched (ER-, PR-, HER2+), and TNBC (ER-, PR-, HER2-) [14,15] based on the results of IHC staining for ER, PR, HER2, and Ki67. The sentinel lymph node (SLN) was marked with methylene blue dye and removed during surgery. The SLN was sliced into 2mm thick sections, and pathological examination was performed to check for metastasis. SLNM was classified according to previous studies, with tumor diameters > 2mm considered macrometastases, tumor diameters > 0.2 mm and ≤ 2mm, or tumor cells < 200 considered micrometastases. Tumor diameters <  0.2 mm or tumor cells < 200 were considered isolated tumor cells [16].

### 2.3. The histopathological evaluation of TILs

**Fig 2:**
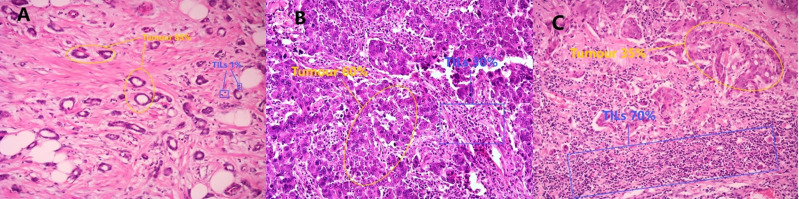
Histopathologic analysis for tumor-infiltrating lymphocyte (TIL) density was performed on a single full-face hematoxylin and eosin-stained tumor section. A: Tumor-infiltrating lymphocyte density is less than 10% (TILs < 10%) (Tumour percentage is 85%, and the TILs score is 1%) (H&E ×  200). B: Tumor-infiltrating lymphocyte density is greater than or equal to 10% and less than 50% (10% ≤ TILs < 50%) (Tumour percentage is 60%, and TILs score is 30%) (H&E ×  200). C: Tumor-infiltrating lymphocyte density is greater than or equal to 50% (TILs ≥ 50%) (Tumour percentage is 35%, and TILs score is 70%) (H&E ×  200).

The histopathological assessment of TILs density in core needle biopsy specimens allows for the categorization of TILs into intratumoral tumor-infiltrating lymphocytes (iTILs) and stromal tumor-infiltrating lymphocytes (sTILs) based on their spatial distribution. iTILs are lymphocytes that are in direct contact with tumor cells and are located within the tumor nest, while sTILs are found within the fibrous stroma of the tumor. Both types have clinical significance. Currently, sTILs are considered to have a higher clinical application value due to their relatively higher quantity, ease of observation and assessment, and higher reproducibility, which is why the International TILs Working Group recommends assessing sTILs [17]. In this study, only sTILs were evaluated, and unless otherwise specified, TILs refer to sTILs in the following text. The Breast Cancer TILs scoring guidelines published by the International Breast Cancer TILs Research Group in 2014 [18], along with subsequent updates and content supplements in 2017 and 2018 [19], provide a standard reference for evaluation. According to the scoring guidelines and previous research, a TIL density of ≥ 10% is defined as high TILs, and a TIL density of < 10% is defined as low TILs. Additionally, breast cancers with a TIL density of ≥ 50% are defined as lymphocyte predominant breast cancer (LPBC), and those with a TIL density of < 50% are defined as non-lymphocyte predominant breast cancer (nLPBC) [20,21](Fig 2).

### 2.4. Statistical analysis

Statistical analysis was performed using SPSS 17.0 software. Descriptive statistics were represented by frequency and percentage (n, %). The t-test was used to compare the distribution of TILs in different sentinel lymph node metastasis states. The correlation between categorical variables based on clinical and pathological characteristics was assessed using the Pearson chi-square test and Fisher’s exact test. Univariate analysis was used to examine the correlation between each variable and SLNM, and factors with a P-value of less than 0.05 in the univariate analysis were further subjected to multivariate logistic regression analysis to calculate the odds ratio (OR) and the 95% confidence interval (CI), with a P-value of less than 0.05 considered as an independent influencing factor. The ROC curve was used to evaluate the value of TILs density in predicting SLNM. The nomogram was constructed using the “nomogram” function in the R programming language.

### 2.5. Ethics approval and consent to participate

This study complies with the provisions of the Declaration of Helsinki in 2013 and has been granted exemption from informed consent by the Ethics Committee of Weifang People’s Hospital (KYLL20240425-1).

## 3. Results

### 3.1. Clinicopathological features

The study included a total of 337 patients for analysis, with a median age of 55 years (range 27-81 years). There were 230 postmenopausal patients (68.2%). There were 179 patients with cT1 (53.1%) and 158 patients with cT2 (46.9%). ER positivity was found in 287 cases (85.2%), PR positivity in 271 cases (80.4%), HER2 positivity in 57 cases (16.9%), and high Ki67 expression in 256 cases (76.0%). Androgen Receptor (AR) positivity was noted in 319 cases (94.7%), and P53 positivity in 256 cases (76.0%), with 118 cases (35.0%) having a nuclear grade of III. There were 79 cases of Luminal A (LA) type (23.4%), 213 cases of Luminal B (LB) type (63.2%), 16 cases of HER2-enriched type (4.7%), and 29 cases of triple-negative (TN) type (8.6%). High TILs were present in 283 patients (84.0%), and low TILs in 54 patients (16.0%). There were 132 patients (39.1%) with LPBC and 205 patients (60.9%) with nLPBC. Macrometastasis in the sentinel lymph node occurred in 116 patients (34.4%), while 221 patients (65.6%) did not have macrometastasis or only had micrometastasis in the sentinel lymph node (Table 1).

**Table 1:** Patients’ baseline characteristics.

Parameters	Number of patients (n = 337) (%)
Age (years old)	Median 55 (range 27-81)
Menstruation (Yes/No)	107 (31.8)/230 (68.2)
Tumor location (Upper outer/ Lower outer/ Upper inner/Lower inner)	181 (53.7)/59 (17.5)/81 (24.0)/16 (4.7)
cT(T1/T2)	179 (53.1)/158 (46.9)
ER(Positive/Negative)	287 (85.2)/50 (14.8)
PR(Positive/Negative)	271 (80.4)/66 (19.6)
Her-2 (Positive/Negative)	57 (16.9)/280 (83.1)
Ki67 (≤14%/ > 14%)	81 (24.0)/256 (76.0)
AR(Positive/Negative)	319 (94.7)/18 (5.3)
P53 (Positive/Negative)	256 (76.0)/81 (24.0)
Nuclear grade(Ⅰ/Ⅱ/Ⅲ/)	11 (3.3)/208 (61.7)/118 (35.0)
LA/LB/HER2 enriched/TNBC	79 (23.4)/213 (63.2)/16 (4.7)/29 (8.6)
TILs (<10%/10-49%/ ≥ 50%)	54 (16.0)/151 (44.8)/132 (39.2)
SLNB (pN0-1mic/pN1-2)	221 (65.6)/116 (34.4)

ER estrogen receptor; PR progesterone receptor; Her-2 human epidermal growth factor receptor 2; AR Androgen Receptor; LA Luminal A (ER+, PR+/-, HER2-, low Ki67); LB Luminal B (ER+, PR+/-, HER2+ or ER+, PR+/-, HER2-, high Ki67); HER2 enriched (ER-, PR-, HER2+); TNBC (ER-, PR-, HER2-); TILs Tumor-infiltrating Lymphocytes; SLNB Sentinel lymph node biopsy.

### 3.2. The relationship between clinical and pathological features and SLNM

The correlation between clinical and pathological features and SLNM (Table 2). For all patients, those over 55 years old had a higher rate of SLNM than those 55 years old or younger (P = 0.039); patients with cT2 had a higher rate of SLNM than those with cT1 (P = 0.016); patients with ER positivity had a higher rate of SLNM than those with ER negativity (P = 0.023); patients with AR positivity had a higher rate of SLNM than those with AR negativity (P = 0.040). Additionally, TILs were significantly correlated with SLNM, with patients with nLPBC having a higher rate of SLNM than those with LPBC (P = 0.010); patients with high TILs had a significantly lower rate of SLNM than those with low TILs (P < 0.001). For patients with LA type breast cancer, SLNM was significantly correlated with age (P = 0.048), menstrual status (P = 0.033), and TILs, with patients with high TILs having a significantly lower rate of SLNM than those with low TILs (P = 0.001). For patients with LB type breast cancer, those with nLPBC had a higher rate of SLNM than those with LPBC (P = 0.035); patients with high TILs had a significantly lower rate of SLNM than those with low TILs (P = 0.001). For patients with TNBC, those with high TILs and LPBC had a lower rate of SLNM (P = 0.003, P = 0.034). However, for patients with HER2 enriched breast cancer, there was no significant statistical correlation between clinical and pathological features and SLNM.

**Table 2:** The relationship between sentinel lymph node metastasis and clinical pathological features in patients.

Parameters	All patients(n = 337) (%)			Luminal A (n = 79 ) (%)			Luminal B (n = 213) (%)			HER2 enriched (n = 16) (%)			TNBC (n = 29) (%)		
	pN0-1mic (n = 221)	pN1-2 (n = 116)	P value	pN0-1mic (n = 57)	pN1-2 (n = 22)	P value	pN0-1mic (n = 129)	pN1-2 (n = 84)	P value	pN0-1mic (n = 13)	pN1-2 (n = 3)	P value	pN0-1mic (n = 22)	pN1-2 (n = 7)	P value
Age (years old)															
≤55	103 (46.6)	68 (58.6)	0.039	27 (47.4)	16 (72.7)	0.048	58 (45.0)	45 (53.6)	0.262	7 (53.8)	2 (66.7)	0.687	11 (50.0)	5 (71.4)	0.410
>55	118 (53.4)	48 (41.4)		30 (52.6)	6 (27.3)		71 (55.0)	39 (46.4)		6 (46.2)	1 (33.3)		11 (50.0)	2 (28.6)	
Menstruation															
Yes	64 (29.0)	43 (37.1)	0.140	15 (26.3)	12 (54.5)	0.033	39 (30.2)	28 (33.3)	0.653	5 (38.5)	0 (0.0)	0.509	5 (22.7)	3 (42.9)	0.357
No	157 (71.0)	73 (62.9)		42 (73.7)	10 (45.5)		90 (69.8)	56 (66.7)		8 (61.5)	3 (100.0)		17 (77.3)	4 (57.1)	
Tumor location															
Upper outer	116 (52.5)	65 (56.0)	0.732	28 (49.1)	12 (54.5)	0.546	66 (51.2)	46 (54.8)	0.906	7 (53.8)	0 (0.0)	0.331	15 (68.2)	7 (100.0)	0.402
Lower outer	37 (16.7)	22 (19.0)		7 (12.3)	4 (18.2)		27 (20.9)	17 (20.2)		2 (15.4)	1 (33.3)		1 (4.5)	0 (0.0)	
Upper inner	57 (25.8)	24 (20.7)		18 (31.6)	6 (27.3)		31 (24.0)	17 (20.2)		3 (23.1)	1 (33.3)		5 (22.7)	0 (0.0)	
Lower inner	11 (5.0)	5 (4.3)		4 (7.0)	0 (0.0)		5 (3.9)	4 (4.8)		1 (7.7)	1 (33.3)		1 (4.5)	0 (0.0)	
cT															
T1	128 (57.9)	51 (44.0)	0.016	39 (68.4)	13 (59.1)	0.441	71 (55.0)	35 (41.7)	0.068	6 (46.2)	1 (33.3)	0.687	12 (54.5)	2 (28.6)	0.390
T2	93 (42.1)	65 (56.0)		18 (31.6)	9 (40.9)		48 (45.0)	49 (58.3)		7 (53.8)	2 (66.7)		10 (45.5)	5 (71.4)	
ER															
Positive	181 (81.9)	106 (91.4)	0.023	–	–		124 (96.1)	84 (100.0)	0.159	–	–		–	–	
Negative	40 (18.1)	10 (8.6)		–	–		5 (3.9)	0 (0.0)		–	–		–	–	
PR															
Positive	175 (79.2)	96 (82.8)	0.473	–	–		121 (93.8)	75 (89.3)	0.302	–	–		–	–	
Negative	46 (20.8)	20 (17.2)		–	–		8 (6.2)	9 (10.7)		–	–		–	–	
HER2															
Positive	37 (16.7)	20 (17.2)	0.908	–	–		24 (18.6)	17 (20.2)	0.859	–	–		–	–	
Negative	184 (83.3)	96 (82.8)		–	–		105 (81.4)	67 (79.8)		–	–		–	–	
Ki67															
≤14%	59 (26.7)	22 (19.0)	0.140	–	–		1 (0.8)	0 (0.0)	0.419	–	–		1 (4.5)	0 (0.0)	0.566
>14%	162 (73.3)	94 (81.0)		–	–		128 (99.2)	84 (100.0)		–	–		21 (95.5)	7 (100.0)	
AR															
Positive	205 (92.8)	114 (98.3)	0.040	–	–		–	–		–	–		6 (27.3)	5 (71.4)	0.071
Negative	16 (7.2)	2 (1.7)		–	–		–	–		–	–		16 (72.7)	2 (28.6)	
P53															
Positive	172 (77.8)	84 (72.4)	0.285	40 (70.2)	16 (72.7)	0.823	104 (80.6)	62 (73.8)	0.310	10 (76.9)	2 (66.7)	0.712	18 (81.8)	4 (57.1)	0.311
Negative	49 (22.2)	32 (27.6)		17 (29.8)	6 (27.3)		25 (19.4)	22 (26.2)		3 (23.1)	1 (33.3)		4 (18.2)	3 (42.9)	
Nuclear grade															
Ⅰ+Ⅱ	139 (62.9)	80 (69.0)	0.282	56 (98.2)	22 (100.0)	0.532	77 (59.7)	57 (67.9)	0.248	3 (23.1)	1 (33.3)	0.712	3 (13.6)	0 (0.0)	0.557
Ⅲ	82 (37.1)	36 (31.0)		1 (1.8)	0 (0.0)		52 (40.3)	27 (31.2)		10 (76.9)	2 (66.7)		19 (86.4)	7 (100.0)	
TILs															
<50%	123 (55.7)	81 (69.8)	0.010	48 (84.2)	19 (86.4)	0.811	67 (51.9)	56 (66.7)	0.035	6 (46.2)	2 (66.7)	0.522	2 (9.1)	4 (71.4)	0.003
≥50%	98 (44.3)	35 (30.2)		9 (15.8)	3 (13.6)		62 (48.1)	28 (33.3)		7 (53.8)	1 (33.3)		20 (90.9)	3 (28.6)	
TILs															
<10%	19 (8.6)	35 (30.2)	<0.001	3 (5.3)	8 (36.4)	0.001	13 (10.1)	23 (27.4)	0.001	2 (15.4)	1 (33.3)	0.489	1 (4.5)	3 (42.9)	0.034
≥10%	202 (91.4)	81 (69.8)		54 (94.7)	14 (63.6)		116 (89.9)	61 (72.6)		11 (84.6)	2 (66.7)		21 (95.5)	4 (57.1)	

ER estrogen receptor; PR progesterone receptor; Her-2 human epidermal growth factor receptor 2; AR Androgen Receptor; LA Luminal A (ER+, PR+/-, HER2-, low Ki67); LB Luminal B (ER+, PR+/-, HER2+ or ER+, PR+/-, HER2-, high Ki67); HER2 enriched (ER-, PR-, HER2+); TNBC (ER-, PR-, HER2-); TILs Tumor-infiltrating Lymphocytes; SLNB Sentinel lymph node biopsy.

### 3.3. The correlation between clinical and pathological features and TILs

**Fig 3:**
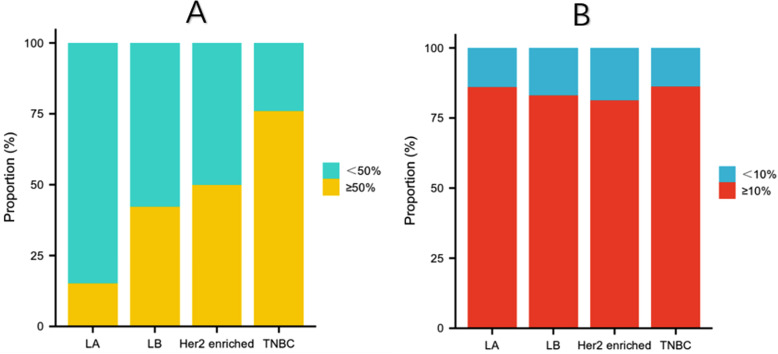
Dividing the density of Tumor-Infiltrating Lymphocytes (TILs) at a threshold of 50% to categorize into lymphocyte predominant breast cancer (LPBC) and non-lymphocyte predominant breast cancer (nLPBC), patients with Triple-Negative Breast Cancer (TNBC) have a higher proportion of LPBC, followed by those with HER2-enriched breast cancer. Patients with Luminal A (LA) and Luminal B (LB) breast cancer have the smallest proportion of LPBC (P <  0.001) (A). When dividing the density of Tumor-Infiltrating Lymphocytes (TILs) with a cutoff of 10% into high TILs and low TILs, there is no significant difference in TILs among Luminal A (LA), Luminal B (LB), HER2-overexpressing, and Triple-Negative (TN) breast cancer types (P = 0.902) (B).

We analyzed the correlation between patient TILs and clinical pathological characteristics (Table 3). When classifying patients into LPBC and nLPBC based on a 50% cutoff value for TILs density, LPBC was significantly associated with the following clinical pathological features: age > 55 years (P = 0.001), amenorrhea (P = 0.023), ER negativity (P < 0.001), PR negativity (P < 0.001), HER2 positivity (P = 0.026), high Ki-67 expression (P < 0.001), AR negativity (P < 0.001), and high nuclear grade (P = 0.026). Upon subgroup analysis, patients with TNBC had a higher proportion of LPBC, followed by those with HER2 enriched breast cancer, while patients with LA and LB types of breast cancer had the smallest proportion of LPBC (P < 0.001) (Fig 3A). However, when analyzing with a 10% cutoff for TILs density to differentiate high TILs from low TILs, apart from age (P = 0.012), no other clinical pathological factors showed a significant correlation with TILs. Upon subgroup analysis, there were also no significant differences in TILs among patients with LA, LB, HER2 enriched, and TN types of breast cancer (P = 0.902) (Fig 3B).

**Table 3:** Patients’ TILs and the relationship with clinical and pathological features.

Parameters	tumor-infiltrating lymphocytes (n = 337)					
	<50% (n = 205)	≥50% (n = 132)	P value	<10% (n = 54)	≥10% (n = 283)	P value
Age (years old)						
≤55	119 (58.0)	52 (39.4)	0.001	36 (66.7)	135 (47.7)	0.012
>55	86 (42.0)	80 (60.6)		18 (33.3)	148 (52.3)	
Menstruation						
Yes	75 (36.6)	32 (24.2)	0.023	23 (42.6)	84 (29.7)	0.079
No	130 (63.4)	100 (75.8)		31 (57.4)	199 (70.3)	
Tumor location						
Upper outer	103 (50.2)	78 (59.1)	0.049	36 (66.7)	145 (51.2)	0.171
Lower outer	33 (16.1)	26 (19.7)		5 (9.3)	54 (19.1)	
Upper inner	60 (29.3)	21 (15.9)		11 (20.4)	70 (24.7)	
Lower inner	9 (4.4)	7 (5.3)		2 (3.7)	14 (4.9)	
cT						
T1	116 (56.6)	63 (47.7)	0.119	32 (59.3)	147 (51.9)	0.373
T2	89 (43.4)	69 (52.3)		22 (40.7)	136 (48.1)	
ER						
Positive	186 (90.7)	101 (76.5)	<0.001	47 (87.0)	240 (84.8)	0.835
Negative	19 (9.3)	31 (23.5)		7 (13.0)	43 (15.2)	
PR						
Positive	179 (87.3)	92 (69.7)	<0.001	43 (79.6)	228 (80.6)	0.853
Negative	26 (12.7)	40 (30.3)		11 (20.4)	55 (19.4)	
HER2						
Positive	27 (13.2)	30 (22.7)	0.026	10 (18.5)	47 (16.6)	0.696
Negative	178 (86.8)	102 (77.3)		44 (81.5)	236 (83.4)	
Ki67						
≤14%	67 (32.7)	14 (10.6)	<0.001	11 (20.4)	70 (24.7)	0.603
>14%	138 (67.3)	118 (89.4)		43 (79.6)	213 (75.3)	
Molecular subtype						
LA	67 (84.8)	12 (15.2)	<0.001	11 (13.9)	68 (86.1)	0.902
LB	123 (57.7)	90 (42.3)		36 (16.9)	177 (83.1)	
HER2 enriched	8 (50.0)	8 (50.0)		3 (18.8)	13 (81.2)	
TNBC	7 (24.1)	22 (75.9)		4 (13.8)	25 (86.2)	
AR						
Positive	202 (98.5)	117 (88.6)	<0.001	52 (96.3)	16 (5.7)	0.748
Negative	3 (1.5)	15 (11.4)		2 (3.7)	267 (94.3)	
P53						
Positive	157 (76.6)	99 (75.0)	0.794	40 (74.1)	216 (76.3)	0.730
Negative	48 (23.4)	33 (25.0)		14 (25.9)	67 (23.7)	
Nuclear grade						
Ⅰ+Ⅱ	143 (69.8)	76 (57.6)	0.026	34 (63.0)	185 (65.4)	0.757
Ⅲ	62 (30.2)	56 (42.4)		20 (37.0)	98 (34.6)	
SLNB						
pN0-1mic	123 (55.7)	98 (74.2)	0.010	19 (35.2)	202 (71.4)	<0.001
pN1-2	81 (44.3)	34 (25.8)		35 (64.8)	81 (28.6)	

ER estrogen receptor; PR progesterone receptor; Her-2 human epidermal growth factor receptor 2; AR Androgen Receptor; LA Luminal A (ER+, PR+/-, HER2-, low Ki67); LB Luminal B (ER+, PR+/-, HER2+ or ER+, PR+/-, HER2-, high Ki67); HER2 enriched (ER-, PR-, HER2+); TNBC (ER-, PR-, HER2-); TILs Tumor-infiltrating Lymphocytes; SLNB Sentinel lymph node biopsy.

### 3.4. Analysis of predictive factors for SLNM

**Fig 4:**
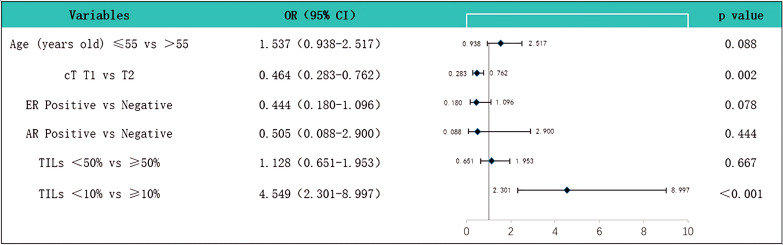
Based on the multivariate analysis, a forest plot was constructed, showing that clinical T stage (P = 0.002, OR = 0.464) and the level of Tumor-Infiltrating Lymphocytes (TILs) (P < 0.001, OR = 4.549) are independent predictive factors for Sentinel Lymph Node Metastasis (SLNM).

**Fig 5:**
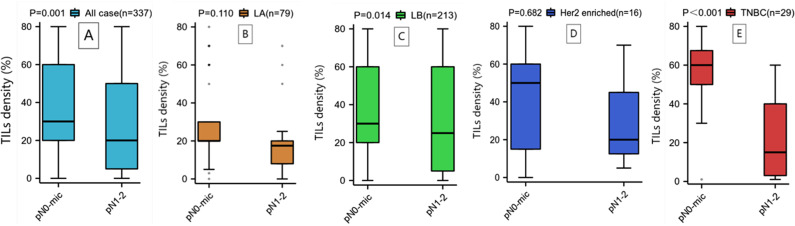
Using t-tests and box plots to analyze the correlation between TILs (Tumor-Infiltrating Lymphocytes) density and SLNM (Sentinel Lymph Node Metastasis), it was found that in all patients, those with SLNM had a significantly lower TILs density than those without SLN metastasis (P = 0.001). (A). When analyzing each subtype, only Luminal B (LB) breast cancer (P = 0.014) and Triple-Negative Breast Cancer (TNBC) (P < 0.001) showed statistical differences (C, E), while other subtypes did not exhibit significant differences (B, D).

**Fig 6:**
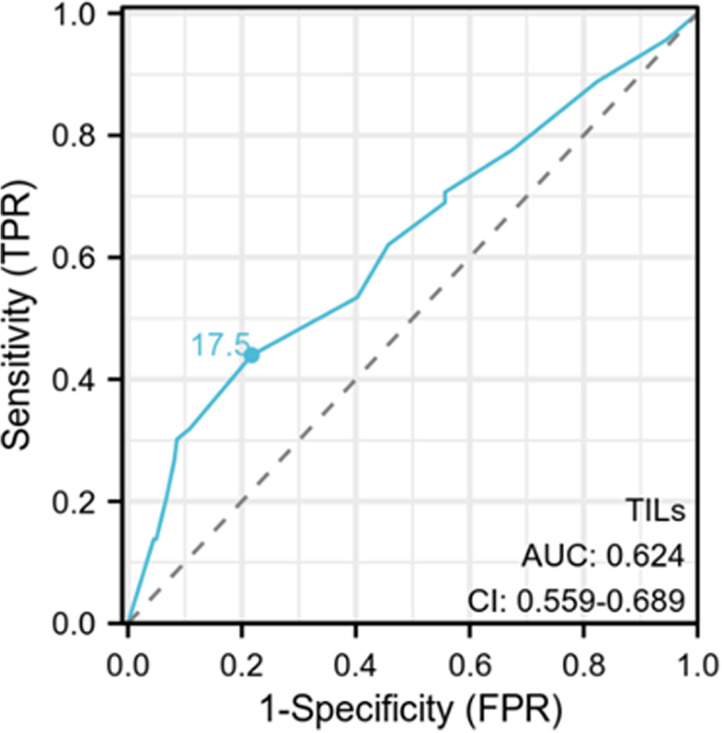
Using the ROC (Receiver Operating Characteristic) curve to evaluate the value of TILs (Tumor-Infiltrating Lymphocytes) density in predicting Sentinel Lymph Node Metastasis (SLNM) in early-stage (cT1-2N0) breast cancer, the AUC (Area Under the Curve) was 0.624 (CI: 0.559-0.689), with a sensitivity of 0.440 and a specificity of 0.783. The optimal cutoff value was 17.5%, indicating good predictive performance.

**Fig 7:**
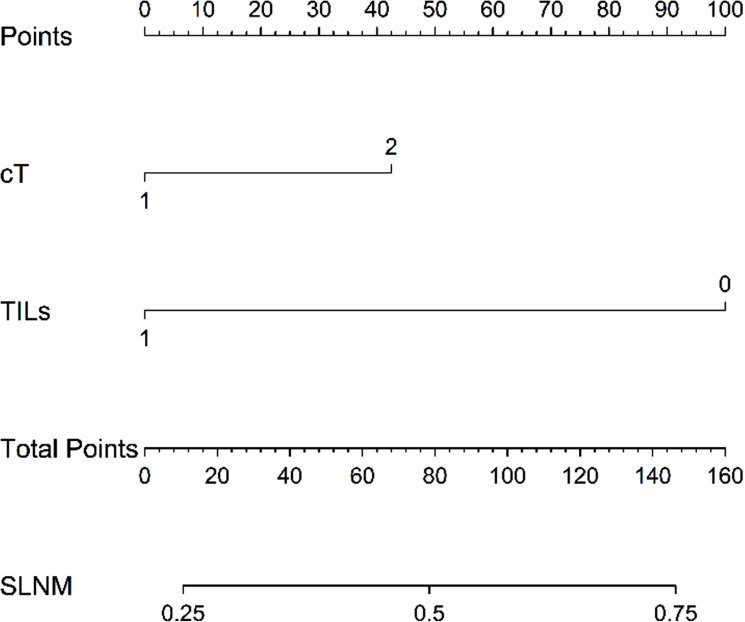
Based on the results of multivariate regression analysis, a nomogram model for Sentinel Lymph Node Metastasis (SLNM) was constructed using the R programming language.

We conducted univariate and multivariate analyses of the predictive factors for SLNM, and the results showed that cT staging (P = 0.002, OR = 0.464) and the level of TILs (P < 0.001, OR = 4.549) are independent predictive factors for SLNM (Table 4) (Fig 4). Upon subgroup analysis (Table 5), in TNBC, LPBC (P = 0.036, OR = 20.000) is an independent predictive factor for SLNM. In LA and LB types of breast cancer, the level of TILs (P = 0.005, OR = 8.895; P = 0.010, OR = 2.895) is an independent predictive factor for SLNM. Additionally, we used t-tests and box plots to analyze the correlation between TILs density and SLNM. In all patients, the TILs density in those with SLNM was significantly lower than those without SLN metastasis (P = 0.001) (Fig 5A). When analyzed by each subtype, only LB type breast cancer (P = 0.014) and TNBC (P < 0.001) showed statistical differences (Fig 5C and 5E), while other subtypes showed no significant differences (Fig 5B and 5D). The ROC curve was used to assess the value of TILs density in predicting SLNM in early-stage (cT1-2N0) breast cancer. The results showed that the AUC was 0.624 (CI: 0.559-0.689), with a sensitivity of 0.440 and a specificity of 0.783, and the optimal cutoff value was 17.5%, indicating that TILs density has good efficacy in predicting SLNM (Fig 6). Based on the results of the multivariate regression analysis, a nomogram model for SLNM was constructed using the R programming language (Fig 7), where the individual scores (Points) for each variable on the left side of the figure are found by drawing a vertical line upward, and the total score (Total Points) is obtained by summing these individual scores; by drawing a vertical line downward from the total score, the corresponding SLNM rate is found, which represents the predicted probability of SLNM for a specific breast cancer patient.

**Table 4:** Univariate and multivariate analysis of sentinel lymph node metastasis in early breast cancer patients.

Parameters	Univariate analysis			multivariate analysis				
	OR	95% CI	p value	B	Wals	OR	95% CI	p value
Age (years old) ≤ 55 vs > 55	0.616	0.391-0.970	0.039	0.430	2.915	1.537	0.938-2.517	0.088
Menstruation yes vs no	1.445	0.898-2.326	0.140					
cT T1 vs T2	1.754	1.115-2.761	0.016	-0.767	9.220	0.464	0.283-0.762	0.002
ER Positive vs Negative	2.343	1.125-4.877	0.023	-0.811	3.103	0.444	0.180-1.096	0.078
PR Positive vs Negative	1.262	0.706-2.256	0.473					
HER2 Positive vs Negative	1.036	0.570-1.883	0.908					
Ki67 ≤ 14% vs > 14%	1.556	0.896-2.702	0.140					
AR Positive vs Negative	4.449	1.005-19.694	0.040	-0.683	0.587	0.505	0.088-2.900	0.444
P53 Positive vs Negative	0.748	0.446-1.253	0.285					
Nuclear grade Ⅰ+Ⅱ vs Ⅲ	0.763	0.473-1.231	0.282					
TILs < 50% vs ≥ 50%	0.520	0.322-0.841	0.010	0.120	0.185	1.128	0.651-1.953	0.667
TILs < 10% vs ≥ 10%	0.218	0.118-0.403	<0.001	1.515	18.965	4.549	2.301-8.997	<0.001

ER estrogen receptor; PR progesterone receptor; Her-2 human epidermal growth factor receptor 2; AR Androgen Receptor; LA Luminal A (ER+, PR+/-, HER2-, low Ki67); LB Luminal B (ER+, PR+/-, HER2+ or ER+, PR+/-, HER2-, high Ki67); HER2 enriched (ER-, PR-, HER2+); TNBC (ER-, PR-, HER2-); TILs Tumor-infiltrating Lymphocytes; SLNB Sentinel lymph node biopsy.

**Table 5:** Univariate and multivariate analysis of sentinel lymph node metastasis in early triple-negative, Luminal A, and Luminal B breast cancer patients.

Parameters	Univariate analysis			multivariate analysis				
	OR	95% CI	p value	B	Wals	OR	95% CI	p value
TNBC								
Age (years old) ≤ 55 vs > 55	0.400	0.063-2.520	0.410					
Menstruation yes vs no	2.550	0.422-15.406	0.357					
cT T1 vs T2	3.000	0.475-18.929	0.390					
Ki67 ≤ 14% vs > 14%	1.333	1.077-1.651	0.566					
AR Positive vs Negative	6.667	1.008-44.097	0.071					
P53 Positive vs Negative	0.296	0.047-1.880	0.311					
Nuclear grade Ⅰ+Ⅱ vs Ⅲ	1.368	1.084-1.728	0.557					
TILs < 50% vs ≥ 50%	0.040	0.004-0.358	0.003	2.996	4.378	20.000	1.209-330.952	0.036
TILs < 10% vs ≥ 10%	0.063	0.005-0.776	0.034	0.405	0.058	1.500	0.055-40.633	0.810
Luminal A								
Age (years old) ≤ 55 vs > 55	0.338	0.115-0.987	0.048	0.158	0.039	1.172	0.244-5.635	0.843
Menstruation yes vs no	3.360	1.205-9.370	0.033	-0.970	1.581	0.379	0.084-1.719	0.209
cT T1 vs T2	1.500	0.543-4.147	0.441					
P53 Positive vs Negative	1.133	0.379-3.393	0.823					
Nuclear grade Ⅰ+Ⅱ vs Ⅲ	0.718	0.625-0.825	0.532					
TILs < 50% vs ≥ 50%	0.842	0.205-3.451	0.811					
TILs < 10% vs ≥ 10%	0.097	0.023-0.415	0.001	2.186	8.020	8.895	1.960-40.372	0.005
Luminal B								
Age (years old) ≤ 55 vs > 55	0.708	0.408-1.299	0.262					
Menstruation yes vs no	1.154	0.640-2.080	0.653					
cT T1 vs T2	1.714	0.983-2.987	0.068					
ER Positive vs Negative	1.667	1.500-1.876	0.159					
PR Positive vs Negative	0.551	0.204-1.490	0.302					
HER2 Positive vs Negative	1.110	0.555-2.219	0.859					
Ki67 ≤ 14% vs > 14%	1.656	1.485-1.847	0.419					
P53 Positive vs Negative	0.677	0.352-1.302	0.310					
Nuclear grade Ⅰ+Ⅱ vs Ⅲ	0.701	0.394-1.250	0.248					
TILs < 50% vs ≥ 50%	0.540	0.306-0.956	0.035	0.302	0.909	1.353	0.727-2.520	0.340
TILs < 10% vs ≥ 10%	0.297	0.141-0.628	0.001	1.063	6.678	2.895	1.293-6.484	0.010

ER estrogen receptor; PR progesterone receptor; Her-2 human epidermal growth factor receptor 2; AR Androgen Receptor; LA Luminal A (ER+, PR+/-, HER2-, low Ki67); LB Luminal B (ER+, PR+/-, HER2+ or ER+, PR+/-, HER2-, high Ki67); HER2 enriched (ER-, PR-, HER2+); TNBC (ER-, PR-, HER2-); TILs Tumor-infiltrating Lymphocytes; SLNB Sentinel lymph node biopsy.

## 4. Discussion

In the era of precision medicine, breast cancer treatment is shifting towards precision, minimally invasive, and personalized approaches. Currently, SLNB has become the standard method for assessing the status of axillary lymph nodes, and numerous studies have demonstrated the high accuracy and predictive value of sentinel lymph node examination [22,23]. For patients with negative sentinel lymph node biopsy results, ALND can be safely avoided. However, whether patients with positive sentinel lymph node biopsy can be exempt from ALND has been the subject of recent clinical trials. The results of the ACOSOG Z0011 clinical trial showed that for patients with cT1-2 tumors, positive in 1-2 sentinel lymph nodes, who underwent breast-conserving surgery, received whole-breast radiotherapy, and had no preoperative treatment, ALND could be omitted without significant differences in 10-year recurrence rates and survival rates compared to patients who underwent axillary dissection [3]. For early-stage breast cancer patients with cN0 sentinel lymph node biopsy positivity, the EORTC 10981-22023 AMAROS study suggested that axillary lymph node radiotherapy could be an alternative to ALND, with comparable 10-year recurrence rates and survival rates, and a significant reduction in the incidence of upper limb edema [5]. In addition, the latest results from the SENOMAC trial (NCT02240472) demonstrated that for clinically lymph node-negative T1, T2, or T3 breast cancer patients with 1-2 sentinel lymph node macro-metastases, who received adjuvant systemic therapy and radiotherapy according to national guidelines, omitting complete ALND was safe, with 5-year overall survival rates of 92.9% and 92.0% [4]. However, some studies have shown that sentinel lymph node biopsy itself may represent an over-treatment for patients with clinically negative axillary lymph nodes [6]. A recent SOUND (Sentinel Node vs Observation After Axillary Ultra-Sound) clinical trial, which included 1,405 patients from multiple centers in a randomized controlled trial, showed no significant difference in 5-year DDFS (1.7% vs. 1.6%) and OS (3.0% vs. 2.6%) between the SLNM group and the group without axillary surgery. These results indicate that axillary surgery can be safely avoided for cT1 breast cancer patients with negative axillary ultrasound findings [7]. Therefore, for early-stage breast cancer patients with clinically negative axillary lymph nodes, finding new markers to predict SLNM is a hot topic in current research. Currently, many studies have shown that the tumor immune microenvironment plays an important role in the occurrence, metastasis, and prognosis of breast cancer and has become one of the new therapeutic targets for breast cancer [24]. TILs are major participants in the tumor immune microenvironment. As early as 1992, researchers first linked breast cancer with TILs. Studies have shown that in breast cancers with high proliferation rates, a large infiltration of lymphocytes seems to improve the recurrence-free survival rate of patients [25]. Since then, research on the relationship between TILs and the development and prognosis of breast cancer has entered a new stage.

Currently, there have been many reports on factors affecting SLNM, such as age, cT stage, pathological nuclear grading, ER, PR, HER2, etc. [26,27]. In our study, the incidence of SLNM was similar to previous reports, with cT stage being one of the predictive factors. However, there have been few reports on the correlation between TILs and SLNM. Previous studies have shown that TILs can serve as predictive factors for lymph node metastasis in early gastric cancer and melanoma [28,29]. In addition, there are also relevant studies reporting that TILs density can be a predictive factor for SLNM in cT1N0 breast cancer [30]. It is worth noting that our study is a study to establish the correlation between TILs and SLNM in early-stage (cT1-2N0) breast cancer and to establish a clinical prediction model. After defining the critical value of TILs density as 10%, we found that breast cancer with high TILs had a lower incidence of SLNM, and TILs and cT stage became independent predictive factors for SLNM. Furthermore, when we analyzed each subgroup, similar to previous reports [31,32], TNBC had the highest TILs density, followed by HER2 enriched breast cancer, with the lowest TILs density in luminal breast cancer. For luminal breast cancer, the relationship between TILs and SLNM was similar to the overall population and also had independent predictive power. However, for TNBC, when divided into LPBC and nLPBC based on a TILs density of 50%, it was clear that LPBC had a lower SLNM rate, and LPBC was an independent predictive factor for SLNM in TNBC. Regrettably, for HER2 enriched breast cancer, due to the insufficient number of cases in our study, no significant results could be drawn.

TNBC has a higher risk of genetic mutations, and studies have shown that for patients with BRCA1 and BRCA2 mutations, TILs have been proven to be a favorable factor for disease-free survival [33]. In terms of overall survival, a 10% increase in TILs density reduces the mortality rate of BRCA1 carriers by 10%, while there is no significant impact on the mortality rate of BRCA2 mutation patients. For patients undergoing neoadjuvant therapy, studies have shown that patients with high TILs have significantly improved pCR rates, DDFS, and OS [34,35]. Our study indicates that in TNBC, TILs have a more significant predictive value for SLNM.

Our study results show the important predictive value of TILs as an indicator of the tumor immune microenvironment (TIME) for the incidence of SLNM in early-stage (cT1-2N0) breast cancer. For other immune cells and factors in TIME, current research has shown that CD8+ T cells, CD4+ T cells, and Foxp3+ regulatory T cells (Tregs) are key to immune surveillance and tolerance [36]. A decrease in the number of CD8+ T cells, an increase in Foxp3+ Tregs, and an increased ratio of Foxp3+ Treg/CD4+ T cells are significantly associated with lymph node metastasis and prognosis [37]. In addition, for TNBC, the presence of PD-L1+ cells also predicts a good prognosis for patients [38]. However, there is little research on the relationship between TIME and SLNM in early-stage breast cancer, which requires further exploration.

It is worth noting that our study has a certain degree of advancement and is a study to establish the correlation between TILs and SLNM in early-stage (cT1-2N0) breast cancer and to establish a clinical prediction model. However, we are also aware of certain limitations in our study. Firstly, as we mentioned earlier, the number of patients with HER2 enriched breast cancer was too small to draw effective conclusions. In addition, our study is a single-center retrospective study with a limited sample size and representativeness, and the established prediction model still needs to be verified through multi-center, large-scale clinical studies in the future.

## 5. Conclusions

Our study shows that the density of TILs and cT stage are independent predictive factors for SLNM in early-stage (cT1-2N0) breast cancer, with significant predictive effects for SLNM in Luminal breast cancer and TNBC.

## Abbreviations

TILs: Tumor-Infiltrating Lymphocytes
SLNM: sentinel lymph node metastasis
ER: estrogen receptor
PR: progesterone receptor;
HER2: human epidermal growth factor receptor 2
IHC: immunohistochemistry
sTILs: interstitial tumor-infiltrating lymphocytes
iTILs: intratumoral tumor-infiltrating lymphocytes
ROC: Receiver Operating Characteristic
AUC: Area Under Curve
SLNB: Sentinel lymph node biopsy
ALND: Axillary Lymph Node Dissection
TAMs: tumor-associated macrophages
FISH: fluorescence in situ hybridization
SLN: sentinel lymph node
LPBC: lymphocyte predominant breast cancer
nLPBC: non-lymphocyte predominant breast cancer
OR: odds ratio
CI: confidence interval
LA: Luminal A
LB: Luminal B
TN: triple-negative
AR: Androgen Receptor
DDFS: Distant disease free survival
OS: Overall Survival
BRCA: Breast Cancer Susceptibility Gene
pCR: pathologic complete response
TIME: tumor immune microenvironment
PD-L1: Programmed cell death 1 ligand 1.
